# Consumption of Chinese Tea-Flavor Liquor Improves Circulating Insulin Levels without Affecting Hepatic Lipid Metabolism-Related Gene Expression in Sprague-Dawley Rats

**DOI:** 10.1155/2013/842343

**Published:** 2013-02-05

**Authors:** Ju-Sheng Zheng, Yuan-Qing Fu, Qi Chen, Tao Huang, Jing Yang, Duo Li

**Affiliations:** ^1^Department of Food Science and Nutrition, Zhejiang University, 866 Yuhangtang Road, Hangzhou 310058, China; ^2^APCNS Centre of Nutrition and Food Safety, 866 Yuhangtang Road, Hangzhou 315200, China

## Abstract

*Objective*. To examine the effect of two Chinese liquors with quite different nonalcoholic components on insulin sensitivity, tissue polyunsaturated fatty acids (PUFA), and hepatic lipid metabolism in SD rats. *Methods*. Thirty-three SD rats were randomized into four groups and maintained in each treatment for 10 weeks: Chinese tea-flavor liquor (TFL, *n* = 9), traditional Chinese liquor (TCL, *n* = 8), ethanol control (EC, *n* = 8), and water control (WC, *n* = 8). *Results*. TFL significantly decreased plasma insulin (*P* = 0.009) and marginally decreased Homeostatic Model Assessment-Insulin Resistance (HOMA-IR) (*P* = 0.05), compared with WC. Hepatic total and n-6 PUFA compositions were significantly decreased in TFL, TCL, and EC groups compared with WC group (*P* < 0.05). TFL significantly increased kidney n-6 PUFA (*P* = 0.05) and total PUFA (*P* = 0.039), compared with EC group. EC group showed significant higher gene expressions of acetyl-CoA carboxylase and steroid response element-binding protein (1c and 2), while there were no significant differences of these gene expressions in TFL or TCL group compared with WC. *Conclusions*. TFL has a beneficial effect on metabolic disorder in relation to improved circulating insulin levels without affecting hepatic lipid metabolism-related gene expressions in rats.

## 1. Introduction

Moderate alcohol consumption (1-2 drinks per day) has been reported to be associated with improved insulin sensitivity [1, 2] and lower risk of cardiovascular disease (CVD) in humans [3, 4]. Red wine is supposed to exert an evident protective effect on cardiovascular system, and nonalcoholic components, such as polyphenols in the red wine, might play a significant role in its protection effects [5–7]. In addition, alcohol could decrease tissue polyunsaturated fatty acids (PUFA) including C20:4n-6 and C22:6n-3 in animal studies [8–11], while this decrease could be attenuated or even reversed by red wine consumption, which might be attributed to the effects of nonalcoholic components [9, 10]. Chinese liquor contains abundant nonalcoholic components [12] and is consumed widely around China. However, to our knowledge, few studies have examined the effects of Chinese liquors on insulin sensitivity and tissue fatty acid compositions in rodent model.

**Figure 1:**
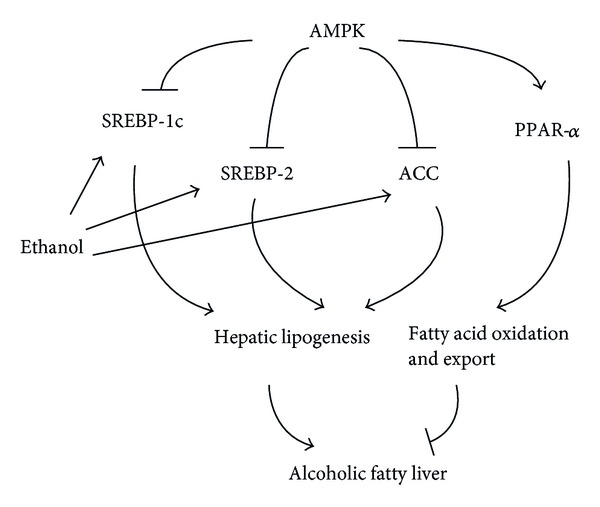
Effect of ethanol treatment on the AMPK-related lipid metabolism pathway. SREBP: steroid response element-binding protein; AMPK: adenosine monophosphate-dependent protein kinase; PPAR-*α*: peroxisome proliferator-activated receptor alpha; ACC: acetyl-CoA carboxylase.

 Hepatic lipids metabolism is mainly controlled by two transcription factors, the peroxisome proliferator-activated receptor-*α* (PPAR-*α*) and the steroid response element-binding protein (SREBP). PPAR-*α* is a receptor for free fatty acids (FFA) and could activate genes involved in transport, oxidation, and export of FFA, while SREBP (SREBP-1c, SREBP-2) is a sensor for cholesterol level and could activate genes involved in synthesis of cholesterol and FFA [13]. In addition, adenosine monophosphate-dependent protein kinase (AMPK) is another key regulator of metabolism, which could drive fatty acid oxidation and export through activation of PPAR-*α* and suppression of SREBP and acetyl-CoA carboxylase (ACC) (Figure 1). Chronic ethanol treatment of cells or animals could activate SREBP and AMPK and inhibit PPAR-*α*, contributing to the potential alcoholic fatty liver [13, 14]. However, whether Chinese liquor would affect the expression of these critical genes in lipid metabolism in the same way as pure ethanol is unclear.

Tea-flavor liquor (TFL) and traditional Chinese liquor (TCL) are two Chinese liquors with abundant but quite different nonalcoholic components. Our previous randomized trial indicates that one-month consumption of both TFL and TCL significantly decreases serum glucose concentrations in healthy young humans [15]. However, whether the effect of these two Chinese liquors on glucose or insulin sensitivity differs from that of pure ethanol is less clear. Therefore, the present study was conducted to test whether daily consumption of TFL and TCL, compared with ethanol control and water control, could affect insulin sensitivity and fatty acid compositions in different tissues of SD rats. In addition, the effect of TFL and TCL treatments on rat hepatic lipid metabolism-related gene expression was examined.

## 2. Methods and Materials

### 2.1. Study Animals and Study Design

Thirty-three male SD rats, three to four months old (518 ± 33.9 g), were purchased from Zhejiang Laboratory Animal Center (Hangzhou, China). The rats were housed in a room under a 12 h light/12 h dark cycle at 22°C. The rats were allowed for acclimatization for three weeks before they were randomized into four groups: a TFL group (*n* = 9), a TCL group (*n* = 8), an ethanol control (EC, *n* = 8) group, and a water control (WC, *n* = 8) group. TFL, TCL, and EC groups received standard rat chow diet together with drinking water containing 3% ethanol in the form of TFL, TCL, and ethanol. WC group received plain water in addition to the standard rat diet. Drinking water, liquors, and rat chow diet were all provided ad lib. The rats were weighed every week, and the amounts of water, liquor and chow diet the rats consumed were measured every day. 10 weeks later, after an overnight fast, all the rats were decapitated and blood was collected in tubes containing ethylenediaminetetra-acetic acid. Plasma was separated from cells by spinning at 2000 rpm for 15 min at 4°C and stored at −20°C if not immediately analyzed. Liver and kidney were removed rapidly, frozen in liquid nitrogen, and stored at −70°C for further analysis. The study protocol was approved by the Ethics Committee of Department of Food Science of Nutrition, Zhejiang University.

### 2.2. Determination of Liquor Composition

TFL and TCL (both 45% (v/v) alcohol) were provided by Guizhou Meijiao Co., Ltd., Guizhou, China. Total acid and ester of the liquors were analyzed by the method issued by the Chinese National Standardization Committee (GB/T 10345-2007). The total polyphenols in the liquors were determined by a colorimetric method using the Folin-Ciocalteu Phenol reagent with gallic acid as equivalent [16]. Briefly, 0.5 mL of supernatant was placed in a 25 mL test tube and mixed with 5 mL Folin-Ciocalteu Phenol reagent. Then 4 mL of Na_2_CO_3_ solution (75 g/L) was added to the tube, and the solution was allowed to stand for 2 hours at room temperature. Absorbance of the blue color complex was measured under 675 nm. Volatile nonalcoholic compounds of the liquors were detected using gas chromatographic together with flame ionization detection. A DB-FFAP capillary column (30 m × 0.25 mm id, 0.25 *μ*m film thickness, J&W, USA) was used. The oven temperature was initially set at 50°C for 6 minutes, then increased to 240°C at 4°C/min, and held for 5 minutes, and nitrogen was used as carrier gas. Butyl acetate was used as the internal standard.

### 2.3. Determination of Plasma Parameters

Total cholesterol (TC), triacylglycerol (TG), high-density lipoprotein cholesterol (HDL-C), low-density lipoprotein cholesterol (LDL-C), alanine aminotransferase (ALT), aspartate aminotransferase (AST), alkaline phosphatase (ALP), creatinine, uric acid, urea nitrogen, and glucose were analyzed on HITACHI 7020 chemistry analyzer using colorimetric test supplied by Diasys Diagnostic Systems (Shanghai) Co., Ltd. Plasma apolipoprotein A1 (apoA1), apolipoprotein B (apoB), and tumor necrosis factor-*α* (TNF-*α*); insulin, interleukin-6 (IL-6), and adiponectin were detected using ELISA (R&DSystems). Homeostatic Model Assessment-Insulin Resistance (HOMA-IR) was calculated as (fasting plasma insulin (in mU/L) × fasting plasma glucose (in mmol/L))/22.5.

### 2.4. Determination of Plasma, Kidney, and Liver Total Fatty Acid Compositions

The total lipid contents of plasma, kidney, and liver were extracted with solvents, and the fatty acid methyl esters were prepared and separated by gas-liquid chromatography as described previously [17]. 

### 2.5. Determination of Lipid Metabolism-Related mRNA Expressions

Total RNA from liver was extracted with Trizol reagent, and reverse transcription was performed in a total volume of 20 *μ*L. After denaturing at 95°C for 5 min, the RT products were preceded for real-time RT PCR using SYBR Green RT-PCR kit (TaKaRa Biotechnology (Dalian) Co., Ltd., China) using Bio-Rad's ICycler IQ Fluorescent Quantity RCR Detecting System. Primers used were listed in Table 1.

Comparative *C*_*T*_ method was used to quantitate gene expression. The amount of target, normalized to a housekeeping gene (*β*-actin) and relative to the control group (water control), was given by the formula 2^−ΔΔ*C*_*T*_^ [18]. 

### 2.6. Statistical Analyses

Data analyses were performed using SPSS 16.0 (SPSS Inc., Chicago, IL, USA). One-way analysis of variance and Tukey's post hoc test were used for statistical analyses. *P* < 0.05 was considered as statistically significant. All the data were expressed as mean ± SD.

## 3. Results

### 3.1. Analyses of Chinese Liquor

Total organic acid was 0.22 g/L and 0.83 g/L for TFL and TCL, respectively, and total ester was 1.58 g/L and 2.64 g/L for TFL and TCL. Ethyl lactate (0.76 g/L). Ethyl hexanoate (0.70 g/L) and isoamylol (0.27 g/L) were the most abundant volatile compounds in TFL, while ethyl hexanoate (1.20 g/L), ethyl lactate (0.71 g/L), and acetic acid (0.63 g/L) in TCL. Total polyphenol was 2.45 mg/L in TFL and 1.88 mg/L in TCL.

### 3.2. Food Consumption, Body Weight Gain, and Plasma Parameters of SD Rats

No significant difference for body weight gain was observed among the four groups, although food consumption in WC group was significantly higher than that of the other three groups (Table 2).

As is shown in Table 3, LDL-C in TFL group was significantly higher than in TCL group (*P* = 0.04), while apoB in TFL group was significantly higher than in EC group (*P* = 0.004). However, there was no significant difference for blood lipids in TFL and TCL groups, compared with WC group. Plasma creatinine was higher in TFL (*P* = 0.02) and TCL (*P* = 0.049) groups than in WC group. ALP in EC group was significantly lower than in WC group (*P* = 0.048).

### 3.3. Plasma Insulin, Glucose, and HOMA-IR

**Figure 2:**
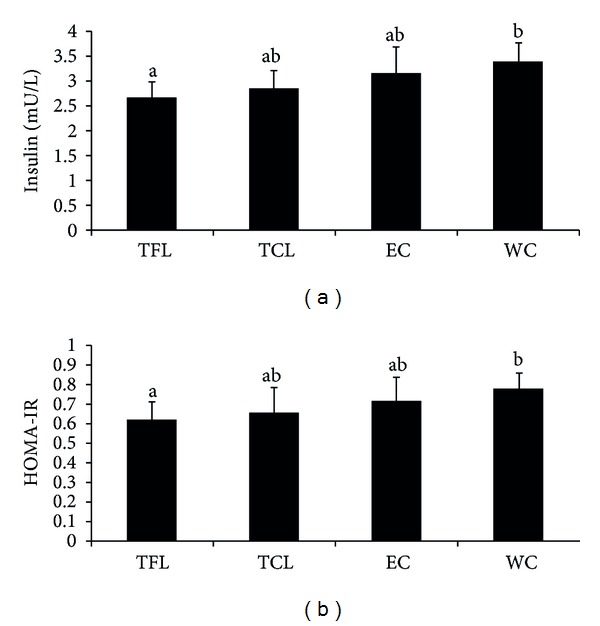
TFL and TCL consumption and plasma insulin and HOMA-IR levels. (a) Effect of TFL and TCL consumption on plasma insulin; (b) Effect of TFL and TCL consumption on HOMA-IR. TFL: tea-flavor liquor; TCL: traditional Chinese liquor; EC: ethanol control; WC: water control.

TFL consumption significantly decreased fasting plasma insulin compared with WC (*P* = 0.009), while TCL nonsignificantly decreased insulin compared with WC (*P* = 0.077) (Figure 2(a)). There was no significant difference for plasma glucose among the four groups. TFL consumption marginally decreased HOMA-IR compared with WC (*P* = 0.05) (Figure 2(b)).

### 3.4. Fatty Acid Composition of Plasma, Kidney, and Liver

For plasma total fatty acid compositions (Table 4), TCL group showed lower total SFA composition than WC group (*P* = 0.015), while total MUFA composition in TFL group was lower compared with EC group (*P* = 0.031). N-6 PUFA (*P* = 0.05) and total PUFA (*P* = 0.039) contents of kidney were significantly higher in TFL group than in EC group (Table 5). TFL (*P* = 0.006) and EC (*P* = 0.014) groups showed significant increases in hepatic total SFA composition compared with WC group, and TFL group also had a higher liver MUFA composition than WC group (*P* = 0.041). N-6 PUFA, C20:5n-3 + C22:6n-3, n-3 PUFA, and total PUFA contents of liver were all lower in TFL, TCL, and EC groups than in WC group; total and n-6 PUFA compositions were significantly lower in TFL, TCL, and EC groups compared with WC group (*P* < 0.05) (Table 6).

### 3.5. Lipid Metabolism-Related Gene mRNA Expression

**Figure 3:**
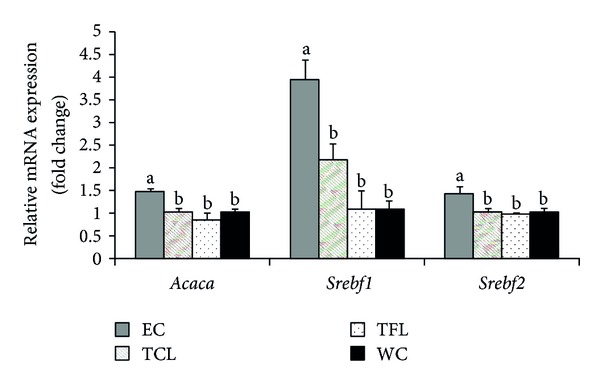
Relative mRNA expressions of key lipid metabolism-related genes. *Acaca*: acetyl-coenzyme A carboxylase alpha; *Srebf1*: sterol regulatory element binding transcription factor 1; *Srebf2*: sterol regulatory element binding transcription factor 2; TFL: tea-flavor liquor; TCL: traditional Chinese liquor; EC: ethanol control; WC: water control.

No significant change was observed for AMPK (encoded by gene *Prkaa1*) and PPAR-*α* (encoded by gene *Ppara*) mRNA expression in TFL, TCL, and EC groups, compared with WC group. However compared with WC group, EC group showed significantly higher mRNA expression of ACC (encoded by gene *Acaca*) (*P* = 0.007), SREBP-1c (encoded by gene *Srebf1*) (*P* < 0.001), and SREBP-2 (encoded by gene *Srebf*2) (*P* = 0.048) (Figures 1 and 3), while there was no significant difference in TFL or TCL group for all these gene expressions compared with WC group.

## 4. Discussion

Neither TFL nor TCL exerted any significant effect on plasma lipids compared with WC. TFL consumption might have a beneficial effect on metabolic disorder in relation to decreased plasma insulin and improved insulin sensitivity. Both TFL and TCL consumption decreased hepatic total and n-6 PUFA compositions compared with WC, while TFL consumption increased total and n-6 PUFA compositions of kidney compared with EC. In addition, compared with WC group, EC group showed significant higher gene expressions of ACC, SREBP-1c, and SREBP-2, which would lead to hepatic fat accumulation, while there was no significant difference of these gene expressions in TFL or TCL group compared with WC.

The protective effects of moderate alcohol consumption against coronary heart disease appeared to be mediated in large part by alcohol-induced increases in HDL-C [19, 20]. However, alcohol consumption in this study did not increase HDL-C level and this might be due to the low ethanol content in drinking liquor and the relatively short alcohol consumption duration in the present study.

Moderate alcohol consumption (1-2 drinks per day) was reported to improve insulin sensitivity in both men and women [2, 21, 22]. Davies et al. [21] concluded that consumption of 30 g/d of alcohol had beneficial effects on insulin and insulin sensitivity in nondiabetic postmenopausal women. Joosten et al. [2] also reported that moderate alcohol consumption for 6 weeks could improve insulin sensitivity in 36 postmenopausal women. In addition, alcohol consumption could promote insulin sensitivity in mice consuming both low-fat and high-fat diets [23]. In the present study, insulin and HOMA-IR in TFL group, but not TCL or EC group, were significantly lower than in WC group. Low and short-term ethanol consumption may contribute to the nonsignificant effects of TCL and EC on insulin sensitivity. However, nonalcoholic components of TFL might also contribute to the improved insulin sensitivity which was not observed in the other groups. Green tea was reported to improve insulin sensitivity [24, 25]; for TFL, nonalcoholic components derived from green tea, one of the fermentation materials, might partially contribute to the improved insulin sensitivity, and the total polyphenols concentration was also higher in TFL than TCL. However, nonalcoholic components taking effect were still to be determined.

Although the alcohol consumption of SD rats in the present study was relatively low (from 2.4 to 2.7 g/d/kg body weight), it was demonstrated that chronic periods of moderate alcohol consumption (from 1.2 to 2.6 g/d/kg body weight) were sufficient to decrease PUFA contents of liver [11, 26, 27]. The results of present study were consistent with previous studies [11, 26, 27], and compositions of total n-3 PUFA, n-6 PUFA, and C20:5n-3 + C22:6n-3 decreased in alcohol group compared with WC group. However, the effect of alcohol on plasma and kidney fatty acids was less evident compared with changes of hepatic fatty acid composition, while TFL could even significantly increase kidney n-6 PUFA and total PUFA content compared with EC. It might be due to that ethanol is mainly metabolized in the liver and ethanol metabolism is capable of generating reactive oxygen species (ROS), which mainly react with PUFA in the liver [11, 28]. In contrast, red wine was reported to preserve kidney long-chain PUFA and nonalcoholic components were reported to contribute to the result [9, 10]. This was consistent with our present findings. TFL could also preserve n-6 and total PUFA contents compared with EC in kidney. And it can be postulated that nonalcoholic components in TFL might partly contribute to the PUFA preservation effect; however, the mechanisms were largely unknown.

Another topic of concern was the fish-like effect of moderate alcohol drinking recently suggested by some researchers [29–31]. Guiraud et al. [30] demonstrated that ethanol drinking would result in a significant increase in plasma docosahexaenoic acid in rats. But the authors did not show changes of fatty acids in liver. As the ethanol intake in that study was much higher than our present study, the PUFA depletion in the liver would be even stronger than the present study. So the fish-like effect of alcohol should be explained with caution as the effect of ethanol on hepatic fatty acid was very sensitive.

Ethanol was reported to increase hepatic lipogenesis by activating SREBP-1 and ACC [32]. Our results were consistent with previous findings that SREBP-1c and ACC mRNA expression were significantly increased in EC group. SREBP-2 is also very important in regulating genes involved in cholesterol metabolism (e.g., HMG-CoA synthase, HMG-CoA reducase) [33]. EC group showed significantly higher SREBP-2 mRNA expressions, which may indicate minor disruption of cholesterol metabolism. However TFL and TCL groups in the present study did not cause any significant change for all these mentioned gene expressions, which might attenuate the impacts of ethanol on lipid metabolism. Given that the ethanol contents in TFL, TCL, and EC groups were the same, nonalcoholic components in TFL and TCL may contribute to the results. The detailed functional nonalcoholic components in the liquors were yet to be determined. However these results should be explained with caution, as the lipid metabolism between humans and rodents is different, and results from rodent model could not be fully extrapolated to humans.

 In conclusion, hepatic total and n-6 PUFA compositions were decreased in all the alcoholic groups, while in kidney, TFL may preserve total and n-6 PUFA compositions. TFL has a beneficial effect for metabolic disorder in relation to improved circulating insulin levels without affecting hepatic lipid metabolism-related gene expressions in rats. Nonalcoholic components may contribute to these results.

## Figures and Tables

**Table 1:** Primers used in the polymerase chain reaction.

Gene	Primers
*Srebf2 *	F: 5′-CGCTCCACAGACCAGGATCA-3′
	R: 5′-TGTCACGAGGCTTTGCACTTG-3′
*Srebf1 *	F: 5′-GGAGCCATGGATTGCACATT-3′
	R: 5′-AGGAAGGCTTCCAGAGAGGA-3′
*Ppara *	F: 5′-GACAAGGCCTCAGGATACCACTATG-3′
	R: 5′-TTGCAGCTTCGATCACACTTGTC-3′
*Prkaa1 *	F: 5′-GGCTCGCCCAATTATGCTG-3′
	R: 5′-AGAGTTGGCACGTGGTCATCA-3′
*Acaca *	F: 5′-CAATCCTCGGCACATGGAGA-3′
	R: 5′-GCTCAGCCAAGCGGATGTAGA-3′
*β*-actin	F: 5′-GGAGATTACTGCCCTGGCTCCTA-3′
	R: 5′-GACTCATCGTACTCCTGCTTGCTG-3′

*Srebf1*: sterol regulatory element binding transcription factor 1; *Srebf2*: sterol regulatory element binding transcription factor 2; *Ppara*: peroxisome proliferator-activated receptor alpha; *Acaca*: acetyl-coenzyme A carboxylase alpha; *Prkaa1*: protein kinase, AMP-activated, alpha 1 catalytic subunit.

**Table 2:** Body weight change and ethanol and food intake of SD rats.

	TFL (*n* = 9)	TCL (*n* = 8)	EC (*n* = 8)	WC (*n* = 8)
Body weight change (kg)	92.3 ± 38.1	116.4 ± 57.5	103.3 ± 50.4	105.5 ± 51.6
Ethanol consumption (g/d/kg body weight)	2.5 ± 0.5	2.4 ± 0.6	2.7 ± 0.5	0
Food consumption (g/d/kg body weight)	39.8 ± 4.4^a^	40.7 ± 5.8^a^	39.3 ± 6.3^a^	47.2 ± 6.6^b^

TFL: tea-flavor liquor; TCL: traditional Chinese liquor; EC: ethanol control; WC: water control; Different letters represent statistically significant difference (*P* < 0.05) between the two groups (e.g., a versus b, b versus c). No significant difference was observed if two groups contain the same letters (e.g., ab versus b, a versus ab).

**Table 3:** Plasma lipids and other parameters after consumption of TFL and TCL.

	TFL (*n* = 9)	TCL (*n* = 8)	EC (*n* = 8)	WC (*n* = 8)
HDL-C (mmol/L)	1.14 ± 0.18	1.08 ± 0.18	1.12 ± 0.18	1.01 ± 0.13
LDL-C (mmol/L)	0.26 ± 0.07^a^	0.18 ± 0.04^b^	0.19 ± 0.04^ab^	0.19 ± 0.09^ab^
TC (mmol/L)	2.07 ± 0.26	1.99 ± 0.30	2.04 ± 0.27	1.89 ± 0.20
LDL/HDL	0.23 ± 0.07	0.17 ± 0.04	0.17 ± 0.03	0.18 ± 0.07
TC/HDL	1.83 ± 0.12	1.85 ± 0.05	1.82 ± 0.07	1.86 ± 0.08
TG (mmol/L)	0.70 ± 0.17	0.74 ± 0.29	0.78 ± 0.18	0.79 ± 0.17
Glucose (mmol/L)	5.40 ± 0.49	5.20 ± 0.62	5.17 ± 0.66	5.26 ± 0.58
ApoA1 (mg/L)	4.48 ± 0.52	4.36 ± 0.61	4.30 ± 0.49	4.52 ± 0.55
ApoB (*μ*g/L)	79.02 ± 5.83^a^	74.67 ± 6.16^ab^	67.69 ± 5.89^b^	73.07 ± 6.80^ab^
Adiponectin (*μ*g/L)	17.51 ± 1.43	16.82 ± 1.37	16.12 ± 1.41	16.87 ± 1.09
IL-6 (ng/L)	21.72 ± 2.25	20.06 ± 1.20	19.94 ± 1.48	20.23 ± 1.98
TNF-*α* (ng/L)	45.60 ± 6.82	44.49 ± 3.99	46.67 ± 4.43	43.49 ± 5.29
Uric acid (*μ*mol/L)	21.36 ± 4.55	21.48 ± 3.19	19.66 ± 3.61	21.34 ± 3.93
Urea nitrogen (mmol/L)	4.18 ± 0.45	4.29 ± 0.88	3.97 ± 0.61	4.43 ± 0.60
Creatinine (*μ*mol/L)	56.66 ± 2.84^a^	56.21 ± 4.23^a^	52.98 ± 2.99^ab^	51.74 ± 2.90^b^
ALT (U/L)	50.22 ± 8.15	53.00 ± 11.65	48.25 ± 7.48	57.12 ± 5.44
AST (U/L)	157.0 ± 31.28	178.75 ± 60.34	169.88 ± 46.53	199.12 ± 70.07
ALP (U/L)	7.33 ± 4.21^ab^	6.88 ± 2.42^ab^	5.25 ± 1.17^a^	9.38 ± 3.16^b^

TFL: tea-flavor liquor; TCL: traditional Chinese liquor; EC: ethanol control; WC: water control; HDL-C: high-density lipoprotein cholesterol; LDL-C: low-density lipoprotein cholesterol; TC: total cholesterol; TG: triacylglycerol; ApoA1: apolipoprotein A1; ApoB: apolipoprotein B; IL-6: interleukin-6; TNF-*α*: tumor necrosis factor-*α*; ALT: alanine aminotransferase; AST: aspartate aminotransferase; ALP: alkaline phosphatase; Different letters represent statistically significant difference (*P* < 0.05) between the two groups (e.g., a versus b, b versus c). No significant difference was observed if two groups contain the same letters (e.g., ab versus b, a versus ab).

**Table 4:** Plasma total fatty acid profiles after consumption of TFL and TCL.

	TFL (*n* = 9)	TCL (*n* = 8)	EC (*n* = 8)	WC (*n* = 8)
14:0	0.44 ± 0.13	0.46 ± 0.11	0.53 ± 0.17	0.50 ± 0.10
15:0	0.33 ± 0.09	0.38 ± 0.09	0.36 ± 0.11	0.36 ± 0.06
16:0	17.30 ± 0.88	16.20 ± 0.99	16.92 ± 1.41	17.83 ± 0.99
18:0	8.27 ± 0.53	7.95 ± 0.62	7.94 ± 0.94	8.57 ± 0.86
20:0	0.41 ± 0.19	0.37 ± 0.06	0.39 ± 0.18	0.28 ± 0.07
SFA	26.76 ± 0.10^ab^	25.37 ± 1.28^a^	26.03 ± 1.26^ab^	27.54 ± 1.14^b^
16:1n7	1.26 ± 0.54	1.02 ± 0.35	1.00 ± 0.13	1.31 ± 0.22
18:1n9	8.19 ± 0.98^a^	8.37 ± 1.53^a^	12.43 ± 4.23^b^	9.01 ± 0.88^a^
18:1n7	1.85 ± 0.30	1.83 ± 0.13	1.82 ± 0.30	1.79 ± 0.17
20:1n9	0.27 ± 0.09^a^	0.56 ± 0.33^b^	0.25 ± 0.18^a^	0.29 ± 0.13^ab^
MUFA	11.57 ± 1.60^a^	11.78 ± 1.78^ab^	15.50 ± 4.41^b^	12.40 ± 1.09^ab^
18:2n6	19.93 ± 2.42	19.86 ± 1.39	20.26 ± 3.63	20.25 ± 1.73
18:3n6	0.94 ± 0.53	1.10 ± 0.59	0.79 ± 0.19	0.63 ± 0.17
20:2n6	0.25 ± 0.07	0.23 ± 0.03	0.22 ± 0.05	0.22 ± 0.01
20:3n6	0.71 ± 0.24	0.67 ± 0.21	0.53 ± 0.08	0.67 ± 0.12
20:4n6	30.00 ± 4.03	29.86 ± 2.34	26.61 ± 7.40	28.88 ± 1.73
22:4n6	0.28 ± 0.04^ab^	0.25 ± 0.05^a^	0.34 ± 0.07^b^	0.28 ± 0.04^ab^
N-6 PUFA	52.11 ± 2.05	51.98 ± 1.47	48.74 ± 5.01	50.94 ± 1.61
18:3n3	1.01 ± 0.35	1.07 ± 0.33	0.86 ± 0.29	1.11 ± 0.20
20:3n3	0.12 ± 0.04	0.13 ± 0.03	0.12 ± 0.07	0.10 ± 0.03
20:5n3	1.80 ± 0.41	2.23 ± 0.62	1.65 ± 0.35	1.69 ± 0.40
22:5n3	0.86 ± 0.17	0.90 ± 0.27	0.92 ± 0.18	0.86 ± 0.11
22:6n3	5.09 ± 0.50	5.28 ± 1.13	4.54 ± 1.03	4.49 ± 0.51
DHA + EPA	6.89 ± 0.72	7.52 ± 1.62	6.20 ± 1.09	6.18 ± 0.60
N-3 PUFA	8.89 ± 1.01	9.61 ± 2.14	8.10 ± 1.46	8.25 ± 0.76
Total PUFA	60.99 ± 1.98	61.59 ± 1.68	56.84 ± 5.97	59.20 ± 1.78
N-3/N-6 PUFA	0.17 ± 0.02	0.19 ± 0.04	0.17 ± 0.03	0.16 ± 0.02

TFL: tea-flavor liquor; TCL: traditional Chinese liquor; EC: ethanol control; WC: water control; SFA: saturated fatty acid; PUFA: polyunsaturated fatty acid; DHA: docosahexaenoic acid; EPA: eicosapentaenoic acid; Different letters represent statistically significant difference (*P* < 0.05) between the two groups (e.g., a versus b, b versus c). No significant difference was observed if two groups contain the same letters (e.g., ab versus b, a versus ab).

**Table 5:** Kidney total fatty acid profiles after consumption of TFL and TCL.

	TFL (*n* = 9)	TCL (*n* = 8)	EC (*n* = 8)	WC (*n* = 8)
14:0	0.48 ± 0.14^a^	0.63 ± 0.18^ab^	0.72 ± 0.25^ab^	0.78 ± 0.15^b^
15:0	0.23 ± 0.03	0.22 ± 0.04	0.20 ± 0.04	0.21 ± 0.03
16:0	19.11 ± 1.01^a^	19.43 ± 0.93^ab^	20.82 ± 1.64^b^	20.76 ± 1.04^bc^
18:0	11.89 ± 1.88	10.30 ± 3.00	9.77 ± 3.74	8.98 ± 2.19
20:0	0.21 ± 0.15	0.10 ± 0.06	0.11 ± 0.04	0.11 ± 0.08
SFA	31.92 ± 2.03	30.68 ± 2.47	31.62 ± 2.08	30.85 ± 2.03
16:1n7	1.72 ± 0.73	1.99 ± 0.59	3.14 ± 1.79	2.70 ± 0.76
18:1n9	11.63 ± 3.46	13.92 ± 4.57	9.77 ± 3.74	8.98 ± 2.19
18:1n7	2.56 ± 0.21	2.55 ± 0.10	2.72 ± 0.65	2.65 ± 0.17
20:1n9	0.14 ± 0.10	0.13 ± 0.06	0.11 ± 0.04	0.12 ± 0.04
MUFA	16.04 ± 4.28	18.59 ± 5.06	22.03 ± 7.76	21.74 ± 4.32
18:2n6	21.10 ± 3.21	24.06 ± 6.53	22.03 ± 4.28	25.26 ± 5.27
18:3n6	0.27 ± 0.10	0.26 ± 0.08	0.23 ± 0.08	0.21 ± 0.08
20:2n6	0.28 ± 0.06	0.26 ± 0.05	0.24 ± 0.10	0.28 ± 0.11
20:3n6	0.79 ± 0.21	0.62 ± 0.24	0.59 ± 0.25	0.51 ± 0.19
20:4n6	22.70 ± 4.50	18.54 ± 7.77	17.26 ± 8.34	15.37 ± 5.77
22:4n6	0.37 ± 0.06	0.30 ± 0.10	0.28 ± 0.12	0.29 ± 0.08
N-6 PUFA	45.52 ± 2.48^a^	44.04 ± 2.77^ab^	40.63 ± 5.86^b^	41.88 ± 2.54^ab^
18:3n3	0.76 ± 0.36	1.26 ± 0.68	1.24 ± 0.59	1.42 ± 0.45
20:3n3	0.18 ± 0.06^a^	0.13 ± 0.07^ab^	0.13 ± 0.08^ab^	0.09 ± 0.04^b^
20:5n3	0.68 ± 0.19	0.63 ± 0.24	0.52 ± 0.16	0.51 ± 0.26
22:5n3	0.63 ± 0.14	0.63 ± 0.29	0.56 ± 0.15	0.54 ± 0.18
22:6n3	2.84 ± 0.62	2.64 ± 1.01	2.20 ± 0.59	2.00 ± 0.75
DHA + EPA	3.53 ± 0.79	3.28 ± 1.23	2.71 ± 0.74	2.51 ± 1.00
N-3 PUFA	5.09 ± 0.61	5.29 ± 1.22	4.63 ± 0.50	4.56 ± 0.88
Total PUFA	50.62 ± 2.90^a^	49.34 ± 2.55^ab^	45.26 ± 5.89^b^	46.44 ± 3.23^ab^
N-3/N-6 PUFA	0.11 ± 0.01	0.12 ± 0.03	0.12 ± 0.02	0.11 ± 0.02

TFL: tea-flavor liquor; TCL: traditional Chinese liquor; EC: ethanol control; WC: water control; SFA: saturated fatty acid; PUFA: polyunsaturated fatty acid; DHA: docosahexaenoic acid; EPA: eicosapentaenoic acid; Different letters represent statistically significant difference (*P* < 0.05) between the two groups (e.g., a versus b, b versus c). No significant difference was observed if two groups contain the same letters (e.g., ab versus b, a versus ab).

**Table 6:** Hepatic total fatty acid profiles after consumption of TFL and TCL.

	TFL (*n* = 9)	TCL (*n* = 8)	EC (*n* = 8)	WC (*n* = 8)
14:0	0.77 ± 0.19^a^	0.59 ± 0.07^ab^	0.77 ± 0.21^a^	0.42 ± 0.07^b^
15:0	0.22 ± 0.06	0.21 ± 0.03	0.26 ± 0.06	0.21 ± 0.02
16:0	25.73 ± 3.12^a^	23.16 ± 1.18^ab^	26.45 ± 3.54^a^	21.84 ± 1.47^b^
18:0	12.92 ± 2.55	11.55 ± 1.79	11.99 ± 2.43	11.51 ± 2.93
20:0	0.07 ± 0.04	0.05 ± 0.01	0.09 ± 0.03	0.04 ± 0.02
SFA	39.71 ± 2.42^a^	35.56 ± 2.45^ab^	39.54 ± 4.39^a^	34.00 ± 3.69^b^
16:1n7	1.22 ± 0.30	1.31 ± 0.52	1.26 ± 0.33	1.00 ± 0.47
18:1n9	12.52 ± 2.87	12.31 ± 2.89	10.34 ± 1.90	9.28 ± 2.27
18:1n7	2.61 ± 0.34	2.73 ± 0.34	2.71 ± 0.44	2.25 ± 0.28
20:1n9	0.21 ± 0.04^a^	0.18 ± 0.04^ab^	0.19 ± 0.06^a^	0.12 ± 0.03^b^
MUFA	16.56 ± 2.79^a^	16.53 ± 3.50^ab^	14.50 ± 2.15^ab^	12.66 ± 2.71^b^
18:2n6	18.62 ± 3.35	20.41 ± 2.87	18.37 ± 3.97	21.85 ± 4.19
18:3n6	0.07 ± 0.03	0.09 ± 0.02	0.09 ± 0.04	0.10 ± 0.04
20:2n6	0.39 ± 0.13	0.40 ± 0.06	0.34 ± 0.04	0.41 ± 0.08
20:3n6	0.63 ± 0.15^ab^	0.70 ± 0.16^ab^	0.58 ± 0.12^a^	0.80 ± 0.16^b^
20:4n6	15.71 ± 3.33	15.63 ± 2.07	17.19 ± 2.29	18.49 ± 3.88
22:4n6	0.31 ± 0.12	0.41 ± 0.12	0.40 ± 0.12	0.49 ± 0.15
N-6 PUFA	35.70 ± 2.07^a^	37.61 ± 3.50^a^	36.94 ± 2.61^a^	42.12 ± 2.48^b^
18:3n3	0.69 ± 0.33	0.76 ± 0.15	0.73 ± 0.23	0.88 ± 0.34
20:3n3	0.10 ± 0.04	0.09 ± 0.04	0.10 ± 0.04	0.10 ± 0.02
20:5n3	0.62 ± 0.31^ab^	0.83 ± 0.29^ab^	0.56 ± 0.12^a^	0.98 ± 0.32^b^
22:5n3	1.18 ± 0.32^a^	1.47 ± 0.37^ab^	1.32 ± 0.24^ab^	1.88 ± 0.60^b^
22:6n3	5.34 ± 0.92^a^	6.35 ± 0.74^bc^	6.07 ± 0.76^ab^	7.22 ± 0.47^c^
DHA + EPA	5.96 ± 0.97^a^	7.17 ± 0.99^bc^	6.63 ± 0.72^ab^	8.20 ± 0.76^c^
N-3 PUFA	7.92 ± 1.21^a^	9.48 ± 1.29^ab^	8.72 ± 0.60^a^	11.05 ± 1.59^b^
Total PUFA	43.61 ± 2.91^a^	47.09 ± 2.86^a^	45.66 ± 2.85^a^	53.17 ± 3.23^b^
N-3/N-6 PUFA	0.22 ± 0.03	0.26 ± 0.06	0.24 ± 0.02	0.26 ± 0.04

TFL: tea-flavor liquor; TCL: traditional Chinese liquor; EC: ethanol control; WC: water control; SFA: saturated fatty acid; PUFA: polyunsaturated fatty acid; DHA: docosahexaenoic acid; EPA: eicosapentaenoic acid; Different letters represent statistically significant difference (*P* < 0.05) between the two groups (e.g., a versus b, b versus c). No significant difference was observed if two groups contain the same letters (e.g., ab versus b, a versus ab).

## Abbreviations

ALT: Alanine aminotransferase
AST: Aspartate aminotransferase
ALP: Alkaline phosphatase
ApoA1: Apolipoprotein A1
ApoB: Apolipoprotein B
AMPK: Adenosine monophosphate-dependent protein kinase
ACAC-*α*: Acetyl-coenzyme A carboxylase alpha
EC: Ethanol control
TCL: Traditional Chinese liquor
HDL-C: High-density lipoprotein cholesterol
IL-6: Interleukin-6
LDL-C: Low-density lipoprotein cholesterol
PUFA: Polyunsaturated fatty acid
PRKA-*α*1: Protein kinase, AMP-activated, alpha 1 catalytic subunit
PPAR-*α*: Peroxisome proliferator-activated receptor alpha
SREBF-1c: Sterol regulatory element binding transcription factor 1c
SREBF-2: Sterol regulatory element binding transcription factor 2
TFL: Tea-flavor liquor
TC: Total cholesterol
TG: Triacylglycerol
TNF-*α*: Tumor necrosis factor-*α*
WC: Water control.
